# Draft Genome Sequence of Thermolongibacillus altinsuensis Strain B1-1, a Novel Hydrogenogenic CO Oxidizer Isolated from Sediment from Lake Biwa in Japan

**DOI:** 10.1128/mra.00334-23

**Published:** 2023-06-05

**Authors:** Jota Suzuki, Yoshinari Imaura, Shiho Nishida, Ryoma Kamikawa, Takashi Yoshida

**Affiliations:** a Laboratory of Marine Microbiology, Graduate School of Agriculture, Kyoto University, Kyoto, Japan; University of Southern California

## Abstract

A facultative anaerobic, thermophilic, hydrogenogenic CO-oxidizing bacterial strain, B1-1, was isolated from a sediment sample from Lake Biwa, a freshwater lake in Japan. B1-1, which is a novel strain of Thermolongibacillus altinsuensis, is capable of hydrogenogenic CO oxidation. Here, we report the draft genome sequence of B1-1 (2.92 Mbp, with a GC content of 41.3%).

## ANNOUNCEMENT

*Thermolongibacillus* is an aerobic, thermophilic, endospore-forming, long rod bacterium (1). In the present study, we isolated a strain of Thermolongibacillus altinsuensis from freshwater sediment that is capable of hydrogenogenic CO oxidation.

The sediment was collected from Lake Biwa (35°13′13″N, 135°59′48″E), a freshwater lake in Japan, at a depth of 72.6 m using an HR-type core sampler (Rigo Co.). In 5 mL of modified B medium (2, 3), 2.5 g of the sediment was incubated at 65°C under 20% CO/80% N_2_. For isolation of the strain, 50 μL of the liquid phase was spread on NBRC802 agar medium and incubated at 65°C under aerobic conditions. A single colony of B1-1 was selected and suspended in modified B medium. After 72 h of incubation under the aforementioned conditions, CO depletion (3.011 μmol/mL) and H_2_ and CO_2_ production (2.975 and 1.031 μmol/mL, respectively) were observed using gas chromatography (Shimadzu).

The partial sequence of the 16S rRNA gene of B1-1 was PCR amplified using the universal primers B27f (5′-AGAGAGTTTGATCCTGGCTCAG-3′) (4) and U515r (5′-TTACCGCGGCKGCTGVCAC-3′) (5). The PCR products were subjected to Sanger sequencing (Eurofins-MWG). A BLASTn search revealed that the sequence of the isolated strain showed the greatest sequence identity (99.13%) to that of T. altinsuensis (GenBank accession number NR_125531.1) (6), suggesting that this strain should be assigned to T. altinsuensis.

After isolation but prior to genome sequencing, the isolate was passaged 4 times in total in the modified B medium or the NBRC802 medium. The genomic DNA was extracted using a DNeasy blood and tissue kit (Qiagen, Valencia, CA, USA). A DNBSEQ-G400 sequencer (MGI Tech Co., Ltd.) was used for 200-bp, paired-end sequencing analysis (Bioengineering Lab. Co., Ltd.), which yielded 20,034,763 paired-end reads.

Default parameters were used for all software unless otherwise specified. Adapter trimming and quality filtering for the generated reads were conducted using Trimmomatic v0.39 (7), setting the SLIDINGWINDOW option as 4:30. The resulting 12,337,528 reads were assembled using SPAdes v3.15.4 (8), and the assembled scaffolds were annotated using the DFAST server v1.2.18 (9).

The draft genome of B1-1 comprised 352 scaffolds, with an average genome coverage of 634×, an *N*_50_ value of 260,943 bp, a total length of 2,923,574 bp, a GC content of 41.3%, and 2,933 coding sequences. The average nucleotide identity (ANI) between strain B1-1 and T. altinsuensis DSM 24979 (GenBank assembly accession number GCA_004341205.1) calculated using JSpeciesWS (10) was 99.08%, which exceeded the 95% threshold for species identity (11). Therefore, strain B1-1 belongs to T. altinsuensis.

The B1-1 strain has one *codh* gene cluster containing *cooS*, which encodes the anaerobic CO dehydrogenase catalytic subunit CooS, the amino acid sequence of which is conserved for the residues constituting the active site (12). The *codh* gene cluster is located adjacent to a gene cluster encoding the membrane-bound hydrogenase (*ech*), implying the contribution of these genes to hydrogenogenic CO oxidation (13). The *codh-ech* gene cluster was not detected in the genome of T. altinsuensis DSM 24979 (GenBank assembly accession number GCA_004341205.1).

### Data availability.

The genome sequence of Thermolongibacillus altinsuensis strain B1-1 has been deposited in the DNA Data Bank of Japan (DDBJ) under the accession number BSVG01000000. Sequence data have been deposited in the DDBJ Sequence Read Archive (DRA) under the accession number DRX440543.
